# The role of acupotomy in treatment of patients with lumbar spinal stenosis: A protocol for a randomized study

**DOI:** 10.1097/MD.0000000000021444

**Published:** 2020-07-31

**Authors:** Yuqin Chen, Huaihua Song, Mingyu Chen, Hua Xu

**Affiliations:** a Department of Rehabilitation, Linyi Central Hospital; b Department of Obstetrics and Gynecology, The third People's Hospital of Linyi; c Department of Neurology, Linyi Central Hospital; d Department of Bone Oncology, Linyi Cancer Hospital, Shandong, China.

**Keywords:** acupuncture, lumbar spinal stenosis, pain control, protocol

## Abstract

**Background:**

Currently, there is some clinical evidence supporting the use of acupuncture in alleviating pain and dysfunction in patients with lumbar spinal stenosis (LSS). However, the question of whether acupuncture could be efficacious for ageing patients remains unanswered. We designed a randomized controlled study to evaluate the safety and feasibility of acupuncture for participants with symptomatic LSS.

**Methods:**

This study is a randomized, single-blind, noninferiority trial. This clinical trial was approved by the Linyi Cancer Hospital. We received informed consent from all patients before surgery. In preparing this report, we adhered to the Consolidated Standards of Reporting Trials guidelines. We randomized consented study participants on a 1:1 ratio to one of two study groups (acupuncture and control groups) using a computer-generated list of random numbers in varying block sizes. Three outcome measures were selected to evaluate the effectiveness and safety of the treatment: visual analogue score and Oswestry disability index, and complicatins. A *P* < .05 was regarded as statistically significant.

**Results:**

The hypothesis was that the acupuncture group would achieve acceptable clinical outcomes as compared to the control group in LSS.

**Trial registration:**

This study protocol was registered in Research Registry (researchregistry5744).

## 1 Introduction

Degenerative lumbar spinal stenosis (LSS) is a highly prevalent condition and causes considerable pain and disability.^[1]^ The underlying somatic anomaly is narrowing of the lumbar spinal canal by bulging discs, hypertrophy of surrounding bone and overgrowth of soft tissues that compromise neural and vascular elements.^[2]^ Patients typically complain of pain in the buttocks and lower extremities provoked by walking or extended standing and relieved by rest or bending forward.^[2–4]^

The primary symptoms of LSS include discomfort, sensory loss, and weakness in the legs, reflecting the involvement of the spinal nerve roots within the lumbar spinal canal. In general, a trial of conservative, nonsurgical treatment precedes surgical treatment. Nonsurgical treatments used for LSS include physical therapy^[5–7]^; analgesic, anti-inflammatory and anticonvulsant medications^[8,9]^; and epidural steroid injections.^[10–12]^ Many patients suffer from the symptoms even while using all of the above mentioned treatments repeatedly.^[13–15]^ In addition to these, patients frequently seek help from complementary and alternative therapy, of which acupuncture is the most popular modality and is gaining increasing use over time.^[16,17]^ It is relatively quick and easy to administer, and allows the patient to self-treat at home as the needles can remain in situ for more than 7 days.^[17]^ Acupuncture in combination with usual care has previously been shown to significantly reduce pain and analgesic intake in postoperative hip and knee surgery, and in chronic low-back pain.^[18]^

Acupuncture is a type of complementary and alternative medicine. It has been accepted worldwide mainly for treatment of acute and chronic pain. However, a previous systematic reviews found no high-quality evidence for or against the effectiveness of acupuncture in patients with LSS.^[19]^ A another meta-analysis found that acupotomy might be beneficial for treating LSS. However, the findings of the meta-analysis should be interpreted cautiously, given the poor methodological quality of the included studies, and potential small-study effects.^[20]^ The lack of quality evidence justifies further randomized trials in this condition to help patients and practitioners make evidence-based decisions with respect to the use of acupuncture in LSS. Therefore, we designed a randomized controlled study to evaluate the safety and feasibility of acupuncture for participants with symptomatic LSS. The hypothesis was that the acupuncture group would achieve acceptable clinical outcomes as compared to the control group in LSS.

## 2 Materials and methods

### 2.1 Study design

This study is a randomized, single-blind, noninferiority trial. This clinical trial was approved by the Linyi Cancer Hospital (SDT10900045) and was registered at the Research Registry (researchregistry5744). We received informed consent from all patients before surgery. In preparing this report, we adhered to the Consolidated Standards of Reporting Trials guidelines.

### 2.2 Participants

Inclusion criteria were the following: participants’ age between 50 and 80 years; degenerative LSS with radiating pain to lower extremities (score of visual analog scale > 4); definite LSS (Schizas grade ≥B) on magnetic resonance imaging; participants who were competent to understand the study protocol; not relieved for at least 3 months by conservative treatments (exercise, analgesics, and epidural steroid injection). Exclusion criteria were the following: congenital stenosis of vertebral canal; serious consequences of LSS (including segmental muscular atrophy, bowl, and bladder disturbances); lumbar tuberculosis; lumbar vertebral tumors; vertebral body compression fracture; spinal instability with annular tears or traction spurs requiring surgery based on flexion-extension lateral views; severe vascular, pulmonary or coronary artery disease that limits ambulation including recent myocardial infarction; cognitive impairment; received medications for pain control during the week prior to baseline assessment.

### 2.3 Randomization and blinding

**Figure 1:**
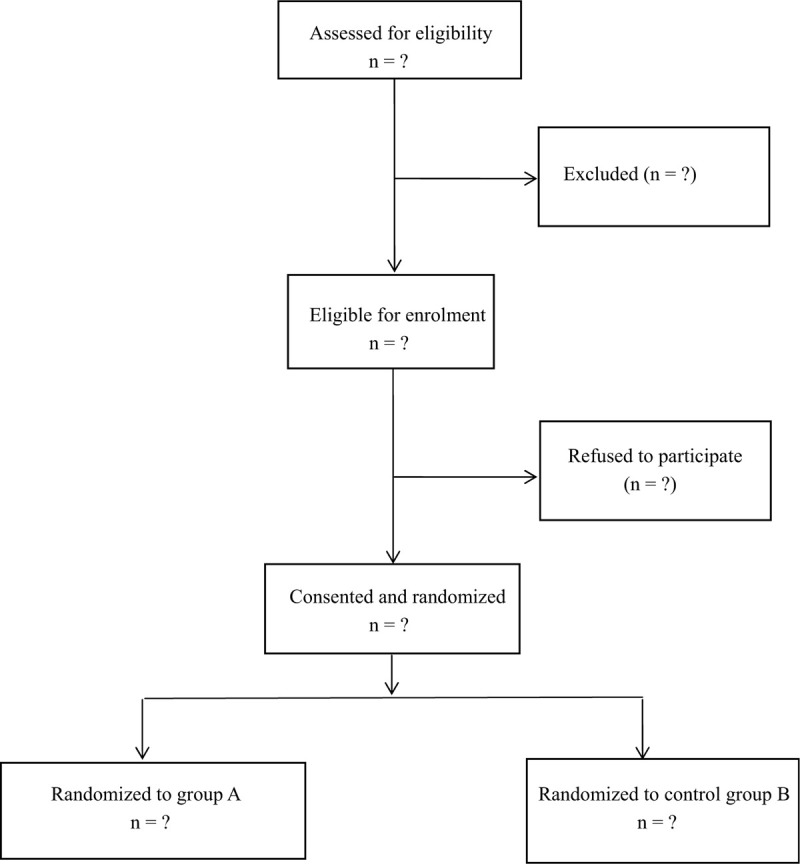
Flow diagram of the study.

We randomized consented study participants on a 1:1 ratio to one of 2 study groups using a computer-generated list of random numbers in varying block sizes. An investigator with no further involvement in the study generated the allocation sequence using the Web site Randomization.com, and concealed the allocation results in sealed opaque sequentially numbered envelopes that were provided to the research coordinator. The acupuncturist who administered the acupuncture had no further role in the study; the acupuncturist, investigator, and nurses were all kept blinded to allocation results (Fig. 1).

### 2.4 Techniques

#### 2.4.1 Acupuncture group

Therapy was conducted by an acupuncturist who had received postgraduate training in acupuncture and had more than 10 years of clinical experience. It was performed 5 times a month (twice in the first week and once each week from 2 to 6) with acupuncture needles 0.18 mm in diameter and 40 mm in length. Five acupoint (GB30, BL40, BL25, BL23, GB34) which are said to be effective for LSS according to the “Korean Medicine Clinical Practice Guideline for Lumbar Herniated Intervertebral Disc in Adults” were chosen. Patients were needled at a depth of approximately 1 cm without any stimulation. BL23, BL25, GB30, BL40, and GB34 on the culpit side were needled first followed by BL25 and BL23 on the contralateral side. The retention time was 20 minutes.

#### 2.4.2 Control group

For those allocated to the control group, optional physical therapies including thermal treatment and interferential current therapy were provided at the participant's request. Concomitant treatments were allowed during the 6-week intervention period. No restrictions were imposed on co-treatment during the post-treatment follow-up period.

### 2.5 Outcomes

Three outcome measures were selected to evaluate the effectiveness and safety of the treatment: visual analogue score (VAS) and Oswestry disability index (ODI), and complications. The VAS is a horizontal line, 100 mm in length, anchored by word descriptors at each end. The patient marks on the line the point that they feel best represent their perception of their current state. The VAS score is determined by measuring in millimeters from the left end to the point that the patient marks. ODI is the principal condition specific outcomes measure used in the management of spinal disorders. The patient questionnaire is composed of topics such as intensity of pain, ability to walk, sit, and stand, sexual function, social life, and sleep quality. Each question is scored on a scale of 0–5 with the first statement being zero and indicating the least amount of disability and the last statement of 5 indicating most severe disability. The index is scored from 0 to 100. Zero is equated with no disability and 100 being maximum disability.

### 2.6 Statistical analysis

All statistical analyses were performed using the SPSS ver. 18.0 (SPSS Inc., Chicago, IL). Values are presented as mean and standard deviation. Patient data were analyzed using the paired t-test. A *P* < .05 was regarded as statistically significant.

## 3 Discussion

Currently, there is some clinical evidence supporting the use of acupuncture in alleviating pain and dysfunction in patients with LSS. However, the question of whether acupuncture could be efficacious for ageing patients remains unanswered. Consequently, we designed a randomized controlled study to evaluate the safety and feasibility of acupuncture for participants with symptomatic LSS. The hypothesis was that the acupuncture group would achieve acceptable clinical outcomes as compared to the control group in LSS. Several limitations of our study should also be acknowledged. First, there is a high risk of bias due to the lack of blinding of the participants and outcome assessors. Second, we did not use a sham-controlled group because we preferred to take a pragmatic approach to assess the overall effectiveness of acupuncture in a ’real world’ clinical scenario. However, this study design is unable to clarify the degree to which specific effects of acupuncture may have contributed to the observed results. Third, the follow-up period was only 3 months, and long-term changes might arguably be more clinically relevant to patients with LSS.

## Author contributions

Yuqin Chen planned the study design and wrote the study protocol. Hua Xu and Huaihua Song reviewed the study protocol. Yuqin Chen and Huaihua Song will recruit participants and collect data. All of the authors have read, commented on, and contributed to the submitted manuscript.

## References

[1] Lurie J Tomkins-Lane C Management of lumbar spinal stenosis. BMJ 2016;352:h6234. 26727925 10.1136/bmj.h6234 PMC6887476

[2] Kreiner DS Shaffer WO Baisden JL An evidence-based clinical guideline for the diagnosis and treatment of degenerative lumbar spinal stenosis (update). Spine J 2013;13:734–43. 23830297 10.1016/j.spinee.2012.11.059

[3] Benoist M The natural history of lumbar degenerative spinal stenosis. Joint Bone Spine 2002;69:450–7. 12477228 10.1016/s1297-319x(02)00429-3

[4] Burgstaller JM Steurer J Gravestock I Long-term results after surgical or non-surgical treatment in patients with degenerative lumbar spinal stenosis: a prospective multi-center study. Spine (Phila Pa 1976) 2020. 10.1097/BRS.0000000000003457 32675604

[5] Mazanec DJ Podichetty VK Hsia A Lumbar canal stenosis: start with nonsurgical therapy. Cleve Clin J Med 2002;69:909–17. 12430977 10.3949/ccjm.69.11.909

[6] Rittenberg JD Ross AE Functional rehabilitation for degenerative lumbar spinal stenosis. Phys Med Rehabil Clin N Am 2003;14:111–20. 12622486 10.1016/s1047-9651(02)00082-7

[7] Bodack MP Monteiro M Therapeutic exercise in the treatment of patients with lumbar spinal stenosis. Clin Orthop Relat Res 2001;384:144–52. 10.1097/00003086-200103000-00017 11249159

[8] Willner S Effect of a rigid brace on back pain. Acta Orthop Scand 1985;56:40–2. 3157290 10.3109/17453678508992977

[9] Whitman JM Flynn TW Childs JD A comparison between two physical therapy treatment programs for patients with lumbar spinal stenosis: a randomized clinical trial. Spine 2006;31:2541–9. 17047542 10.1097/01.brs.0000241136.98159.8c

[10] Roelofs PD Deyo RA Koes BW Nonsteroidal anti-inflammatory drugs for low backpain: an updated Cochrane review. Spine 2008;33:1766–74. 18580547 10.1097/BRS.0b013e31817e69d3

[11] Cuckler JM Bemini PA Wiesel SW The use of epidural steroids in the treatment of lumbar radicular pain. A prospective, randomized, double-blind study. J Bone Joint Surg Am 1985;67:63–6. 3155742

[12] Koes BW Scholten RJ Mens JM Efficacy of epidural steroid injections for low back pain and sciatica: a systematic review of randomized clinical trials. Pain 1995;63:279–88. 8719528 10.1016/0304-3959(95)00124-7

[13] Airaksinen O Brox JI Cedraschi C Chapter 4. European guidelines for the management of chronic nonspecific low back pain. Eur Spine J 2006;15: Suppl 2: S192–300. 16550448 10.1007/s00586-006-1072-1 PMC3454542

[14] Chou R Qaseem A Snow V Diagnosis and treatment of low back pain: a joint clinical practice guideline from the American College of Physicians and the American Pain Society. Ann Intern Med 2007;147:478–91. 17909209 10.7326/0003-4819-147-7-200710020-00006

[15] Xue CC Zhang AL Lin V Acupuncture, chiropractic and osteopathy use in Australia: a national population survey. BMC Public Health 2008;8:105. 18377663 10.1186/1471-2458-8-105 PMC2322980

[16] Hopton AK Curnoe S Kanaan M Acupuncture in practice: mapping the providers, the patients and the settings in a national cross-sectional survey. BMJ Open 2012;2:e000456. 10.1136/bmjopen-2011-000456 PMC3278493 22240649

[17] Liu L Skinner MA McDonough SM Acupuncture for chronic low back pain: a randomized controlled feasibility trial comparing treatment session numbers. Clin Rehabil 2017;31:1592–603. 28459161 10.1177/0269215517705690

[18] Hunter RF McDonough SM Bradbury I Exercise and auricular acupuncture for chronic low-back pain: a feasibility randomized-controlled trial. Clin J Pain 2012;28:259–67. 21753728 10.1097/AJP.0b013e3182274018

[19] Kim KH Kim TH Lee BR Acupuncture for lumbar spinal stenosis: a systematic review and meta-analysis. Complement Ther Med 2013;21:535–56. 24050593 10.1016/j.ctim.2013.08.007

[20] Kwon CY Yoon SH Lee B Acupotomy for the treatment of lumbar spinal stenosis: a systematic review and meta-analysis. Medicine (Baltimore) 2019;98:e16662. 31393365 10.1097/MD.0000000000016662 PMC6708781

